# Clinical Correlation Between Computerized Tomography Findings and Pathologic Diagnosis in Patients Surgically Treated for Complex Renal Cysts in a Colombian Tertiary Center

**DOI:** 10.7759/cureus.6257

**Published:** 2019-11-29

**Authors:** David Castañeda-Millán, Darwin Barros-Valderrama, Diego Camacho-Nieto, Carlos A Riveros, Juan Alzate-Granados, Javier Salgado-Tovar, Wilfredo Donoso-Donoso

**Affiliations:** 1 Urology, Universidad Nacional De Colombia, Bogotá, COL; 2 Urology, Hospital Universitario Mayor - Méderi/ Universidad Del Rosario, Bogotá, COL; 3 Medicine, Universidad Nacional De Colombia, Bogotá, COL; 4 Epidemiology and Public Health, Universidad Nacional De Colombia, Bogotá, COL; 5 Urology, Hospital Universitario Mayor - Méderi/Universidad Del Rosario, Bogotá, COL

**Keywords:** kidney, cysts, tomography, x ray computed, kidney neoplasms

## Abstract

Introduction

Recent data have reexamined the historical rates of malignancy observed in Bosniak IIF and III cystic lesions, and this has led to an endorsement of the use of active surveillance as the standard of care for Bosniak III renal cysts by affirming that overtreatment rates for this subgroup are close to 50%. In light of this, the present study describes the correlation between imaging studies and pathologic diagnosis in patients surgically treated for complex renal cysts in Colombia.

Materials and methods

This is a retrospective, descriptive, and observational study. We analyzed the medical histories of patients who had been surgically treated for complex renal cysts between 2010 and 2018 in the urology department of a tertiary center in Bogota, Colombia. The exclusion criteria were incomplete clinical histories and absent diagnostic images or their official interpretation.

Results

Out of the 235 urological procedures performed, nine were excluded. And 6.19% (n = 14) were cases of surgically treated complex renal cysts; 38.46% were compatible with Bosniak IIF, 46.15% were Bosniak III, and 15.38% were Bosniak IV. The percentage of renal cancer as revealed by the histopathological study was 60, 66.7 and 100 for renal cysts Bosniak IIF, III, and IV, respectively; 77.7% of these confirmed oncological cases had received a diagnosis of clear cell renal carcinoma.

Conclusions

In our study, the percentage of malignancy in patients with renal cysts Bosniak IIF and III was found to be greater compared to the findings in the recent medical literature. We believe that the management offered to the population with complex renal cysts in Colombia should be tailored to the individual risk characteristics of each patient.

## Introduction

Since 1986, the Bosniak radiological classification has become a useful tool for decision making pertaining to patients with renal cystic lesions [1]. In 1993, the IIF group was added to the classification, and since then, in spite of the multiple dilemmas generated around its limitations, there has been an implied understanding that the management of renal cystic lesions depends on the probability of malignancy: low probability (Bosniak I, II, and IIF) vs. high probability (Bosniak III and IV) ) [2,3,4].

For years, clinical practice guidelines have suggested that the management of cystic lesions with a high probability of malignancy should be similar to that of renal masses [5,6]. However, recent studies have demonstrated a more favorable oncological behavior for renal cysts Bosniak III, even reaching the conclusion that this type of lesions could benefit from active surveillance since overtreatment rates have been shown to be as high as 49% [7,8].

In Colombia, there have been no studies that demonstrate the oncological behavior of complex renal cysts. Hence, the present study aims to define and analyze the percentage of malignancy in surgically treated patients for complex renal cysts (Bosniak IIF, Bosniak III, and Bosniak IV) in the urology department of a university hospital in Bogotá, Colombia with the aid of recent global data.

## Materials and methods

We performed a descriptive study of a series of cases by reviewing the clinical records of patients who had undergone renal surgical procedures (open or laparoscopic nephrectomy/heminephrectomy) at the Hospital Universitario Mayor - Mederi in Bogota, Colombia from February 2010 to January 2018. The records were filtered by surgical indication (renal tumor, non-functioning kidney, complex renal cyst, trauma, etc.), and those cases with a surgical indication for the management of complex renal cyst were selected for analysis. A review of the medical record of every case was performed, including the diagnostic images and/or their official radiology reports, along with the results of the histopathological studies. The frequency of the different pathological diagnoses assigned to each patient was evaluated. Cases with incomplete clinical information (demographic data, surgical indication, CT images or their official reports, and pathology results) were excluded from our analysis. The results are expressed in absolute and relative frequencies for the qualitative variables, while the quantitative variables are presented in the form of average measures.

## Results

During the study period, 235 renal surgical procedures were performed (open or laparoscopic nephrectomy/heminephrectomy) in the urology department of the Hospital Universitario Mayor- Mederi. Nine patients were excluded from our analysis as they did not have complete information for the study. Of the remaining 226 patients, 14 (6.19%) were found to have been surgically treated for complex renal cysts. All of these patients had taken a scan of the abdomen and pelvis as part of their imaging studies; only one patient had an MRI performed in addition to CT.

Of the patients who had undergone surgery for the management of complex renal cyst, five (38.46%) presented image findings compatible with Bosniak IIF, six (46.15%) with Bosniak III, and two (15.38%) with Bosniak IV; and one patient was excluded because there was no radiological image or report available (Table 1).

**Table 1:** Characteristics of patients who underwent surgery for complex renal cysts between 2010 and 2018 F: female; M: male; RCC: renal cell carcinoma (clear cell subtype); AHT: arterial hypertension; T2DM: type 2 diabetes mellitus; PKD: polycystic kidney disease; ESRD: end-stage renal disease; RT: renal transplant; UA: unavailable; N/A: not applicable

Age and gender	Bosniak classification	Size of renal cystic lesion, mm	Side affected	Pathologic diagnosis	Fuhrman grade	Medical history	Tumor recurrence
53, F	IIF	30 x 20	Right	Angiomyolipoma	N/A	Smoking	No
52, F	IIF	25 x 25	Right	RCC	2	AHT	Yes
34, M	IIF	113 x 50	Left	Renal cyst with interstitial sclerosis	N/A	AHT, smoking	No
69, M	IIF	80 x 50	Left	RCC	1	T2DM	No
54, M	IIF	76 x 50	Right	RCC	UA	PKD, AHT, ESRD, RT	No
68, M	III	30 x 22	Left	Renal cyst with fibrosclerosis	N/A	T2DM, smoking	No
67, F	III	25 x 30	Right	RCC	3	T2DM, AHT	No
67, F	III	20 x 20	Right	RCC	3	AHT	No
72, M	III	35 x 30	Right	Nonspecific tumoral cystic lesion	UA	AHT, smoking	No
44, M	III	UA	Right	Renal cyst with tubular atrophy	N/A	None	No
66, M	III	30 x 30	Left	Multilocular cystic neoplasm	UA	None	No
65, F	IV	UA	Right	RCC	4	None	No
59, M	IV	95 x 99	Right	RCC	2	AHT	No

Five (38.46%) of the patients were females, and 8 (61.54%) were males. The average age was 59.23 years (range: 34-72 years). The average size of the renal cystic lesion was 50.81 x 38.72 mm. None of the patients had presented metastatic involvement at the time of diagnosis. In the group of patients with imaging lesions classified as Bosniak IIF (n = 5), three (60%) had an oncological diagnosis in the pathology report (100% clear cell renal carcinoma). Within the group corresponding to the Bosniak III classification (n = 6), two cases (33.33%) had been initially classified as Bosniak IIF but had progressed to Bosniak III during the follow-up. In this group, four (66.7%) patients had presented pathological diagnosis after surgical resection (50% clear cell renal carcinoma, 25% multilocular cystic neoplasm, and 25% nonspecific tumoral cystic lesion). In the group corresponding to the Bosniak IV classification, both (100%) patients had received a pathological diagnosis of clear cell renal carcinoma.

Surgical indications in the group of patients with Bosniak IIF renal cysts are described in Table 2. Four (80%) patients undergoing surgical management for Bosniak IIF lesions had received treatment by laparoscopic heminephrectomy. The remaining case was managed by radical laparoscopic nephrectomy. One case had presented a tumor recurrence after three years of follow-up, which had required a new laparoscopic tumorectomy.

**Table 2:** Surgical indications in patients with complex renal cysts Bosniak IIF F: female; M: male

Case (age and gender)	Surgical indication
53, F	Symptomatic angiomyolipoma adjacent to complex renal cyst.
52, F	Desire of the patient not to continue radiological follow-up in addition to lumbar pain.
34, M	Non-functioning duplex kidney + renal cyst Bosniak IIF.
69, M	Progression in radiological follow-up (Bosniak II to IIF).
54, M	Recent history of contralateral renal carcinoma + acquired polycystic kidney disease.

Four (66.7%) patients with complex renal cysts Bosniak III had undergone surgical management by laparoscopic heminephrectomy; the remaining two (33.3%) had undergone radical laparoscopic nephrectomy. All patients with Bosniak IV complex kidney cysts had had laparoscopic radical nephrectomy performed because of the described proximity of the lesions to the interpolar region and the collecting system.

## Discussion

The modified Bosniak radiological classification is an immensely useful tool for surgical decision making pertaining to patients at risk of renal cystic neoplasia. Historically, it has been estimated that between 5 and 10% of cases of renal cancer originate from renal cystic pathology [8,9]. Recently, an update to the Bosniak classification was proposed, with the addition of MRI criteria, with an intention to improve the ability of the original classification to predict the likelihood of malignancy in a cystic renal lesion [10]. In our study, which focused on the group of patients with an indication of surgical management, we found that 6.9% of cases of renal cancer were initially associated with complex renal cysts.

Unlike the global studies conducted in recent years, the percentage of patients with oncological disease was higher in our sampled population [11-17]; 60% and 66.7% of malignancy evidenced in patients with renal cysts Bosniak IIF and III in this study are a far cry from the results recently reported by Schoots et al. (14% and 51%, respectively) [8]. When we performed a comparative analysis against the published literature of recent years, the percentage of malignancy found in our group of patients with renal cysts Bosniak IIF and III was found to be higher (Table 3).

**Table 3:** Comparison of the percentage of malignancy among various studies for complex renal cysts Bosniak IIF and III between 2012 and 2018 *Percentage of malignancy **Unpublished data N/A: not applicable

Author	Year	Bosniak IIF*	Bosniak III*
Castañeda et al.**	2018	60	66.7
Schoots et al. [8]	2017	14	51
Mousessian et al. [13]	2017	N/A	72
Sevcenco et al. [16]	2017	6.7	55.1
Oh et al. [15]	2016	17.1	38
Smith et al. [9]	2015	38	40
Reese et al. [17]	2014	33	66
Weibl et al. [12]	2014	30	64
Hwang et al. [11]	2012	0	83.3

Despite the limitations derived from our sample size, it is necessary to highlight that, of the patients with a reported Fuhrman nuclear grade in this study, 50% of patients presented grades 3 and 4 (high grades). These results contrast with the experiences described by Hwang et al., Mousessian et al., and Donin et al., who described a higher proportion (73%, 91%, 93.7%, respectively) of patients with low-grade Fuhrman grades (1 or 2) in patients with cystic renal carcinoma [11,13,14]. In spite of the significant percentage of patients with high-grade oncological lesions, none of the patients included in this study had metastatic compromise at the time of diagnosis and only one patient had a tumor recurrence. In the group analyzed, of the total number of patients who received an oncological diagnosis by histopathological analysis, 77.7% (n = 9/14) of cases had clear cell renal carcinoma; this finding in terms of frequency is similar to that described in recent literature globally [17].

We are aware that the present study has limitations, mainly related to the size of the sample, the selection of patients who were taken solely to surgical management (selection bias), and the interpretation of the diagnostic images by different radiologists (interobserver variability). But regardless of these limitations, the percentage of patients with complex renal cysts Bosniak IIF and Bosniak III who received oncological diagnosis was found to be higher than the average described in the current medical literature. Therefore, based on the results found, it is not possible to assume that renal cysts Bosniak IIF and III present a low proportion of malignancy in our environment. The results presented here constitute the first study of its kind conducted in Colombia, and demonstrate the need to pay close attention to the risk-benefit ratio generated by any medical intervention in patients with complex renal cysts (active surveillance vs. surgical management).

Thus, the management of complex renal cysts in a local setting must have an individualized approach, which should be adapted to the individual risk characteristics for cancer, and to the possibilities and assurances in providing a strict follow-up for patients with Bosniak IIF and III complex cysts. The findings presented in this article endorse the idea of tailoring a management strategy not very different from that found in current clinical practice guidelines for patients with complex renal cysts (Bosniak III and IV) and localized renal masses. Furthermore, the results call for a stricter periodical follow-up for patients with renal cysts Bosniak IIF given the high percentage of malignancy observed.

## Conclusions

The Bosniak radiological classification is a useful tool for estimating the probability of malignancy in renal cysts. In this study, the percentage of malignancy in patients with renal cysts Bosniak IIF and III was found to be greater than that described in recent literature. The most frequent type of cancer was clear cell renal carcinoma, and no patient had metastatic involvement at the time of diagnosis. The findings demonstrate the need to provide strict monitoring in a local environment for patients with Bosniak IIF renal cysts, and to offer oncological management similar to localized renal masses for patients with renal cysts Bosniak III and IV.
